# In Silico Molecular Docking Analysis of Karanjin against Alzheimer’s and Parkinson’s Diseases as a Potential Natural Lead Molecule for New Drug Design, Development and Therapy

**DOI:** 10.3390/molecules27092834

**Published:** 2022-04-29

**Authors:** Charles Gnanaraj, Mahendran Sekar, Shivkanya Fuloria, Shasank S. Swain, Siew Hua Gan, Kumarappan Chidambaram, Nur Najihah Izzati Mat Rani, Tavamani Balan, Sarah Stephenie, Pei Teng Lum, Srikanth Jeyabalan, M. Yasmin Begum, Vivek Chandramohan, Lakshmi Thangavelu, Vetriselvan Subramaniyan, Neeraj Kumar Fuloria

**Affiliations:** 1 Faculty of Pharmacy and Health Sciences, Royal College of Medicine Perak, Universiti Kuala Lumpur, Ipoh 30450, Malaysia; charles.gnanaraj@unikl.edu.my (C.G.); najihah.izzti@gmail.com (N.N.I.M.R.); tavamani@unikl.edu.my (T.B.); 2 Department of Pharmaceutical Chemistry, Faculty of Pharmacy and Health Sciences, Royal College of Medicine Perak, Universiti Kuala Lumpur, Ipoh 30450, Malaysia; peiteng1013@gmail.com; 3 Faculty of Pharmacy, Centre of Excellence for Biomaterials Engineering, AIMST University, Bedong 08100, Malaysia; 4 Division of Microbiology and NCDs, ICMR-Regional Medical Research Centre, Bhubaneswar 751023, India; swain.shasanksekhar86@gmail.com; 5 School of Pharmacy, Monash University Malaysia, Bandar Sunway 47500, Malaysia; gan.siewhua@monash.edu; 6 Department of Pharmacology, College of Pharmacy, King Khalid University, Abha 62529, Saudi Arabia; kumarappan@kku.edu.sa; 7 School of Biological Sciences, Faculty of Science and Technology, Quest International University Perak, Jalan Raja Permaisuri Bainun, Ipoh 30250, Malaysia; sarah.stephenie@qiu.edu.my; 8 Department of Pharmacology, Sri Ramachandra Faculty of Pharmacy, Sri Ramachandra Institute of Higher Education and Research (DU), Porur, Chennai 600116, India; srikanth.j@sriramachandra.edu.in; 9 Department of Pharmaceutics, College of Pharmacy, King Khalid University, Abha 61421, Saudi Arabia; ybajen@kku.edu.sa; 10 Department of Biotechnology, Siddaganga Institute of Technology, Tumakuru 572103, India; vivek@sit.ac.in; 11 Center for Transdisciplinary Research, Department of Pharmacology, Saveetha Dental College and Hospital, Saveetha Institute of Medical and Technical Sciences, Saveetha University, Chennai 600077, India; lakshmi@saveetha.com; 12 Faculty of Medicine, Bioscience and Nursing, MAHSA University, Jalan SP 2, Bandar Saujana Putra, Jenjarom 42610, Malaysia; drvetriselvan@mahsa.edu.my

**Keywords:** karanjin, Parkinson’s disease, Alzheimer’s disease, in silico, bioinformatics, Lipinski’s rule, molecular dynamics, drug-likeness, ADMET

## Abstract

Parkinson’s disease (PD) and Alzheimer’s disease (AD) are neurodegenerative disorders that have emerged as among the serious health problems of the 21st century. The medications currently available to treat AD and PD have limited efficacy and are associated with side effects. Natural products are one of the most vital and conservative sources of medicines for treating neurological problems. Karanjin is a furanoflavonoid, isolated mainly from *Pongamia pinnata* with several medicinal plants, and has been reported for numerous health benefits. However, the effect of karanjin on AD and PD has not yet been systematically investigated. To evaluate the neuroprotective effect of karanjin, extensive in silico studies starting with molecular docking against five putative targets for AD and four targets for PD were conducted. The findings were compared with three standard drugs using Auto Dock 4.1 and Molegro Virtual Docker software. Additionally, the physiochemical properties (Lipinski rule of five), drug-likeness and parameters including absorption, distribution, metabolism, elimination and toxicity (ADMET) profiles of karanjin were also studied. The molecular dynamics (MD) simulations were performed with two selective karanjin docking complexes to analyze the dynamic behaviors and binding free energy at 100 ns time scale. In addition, frontier molecular orbitals (FMOs) and density-functional theory (DFT) were also investigated from computational quantum mechanism perspectives using the Avogadro-ORCA 1.2.0 platform. Karanjin complies with all five of Lipinski’s drug-likeness rules with suitable ADMET profiles for therapeutic use. The docking scores (kcal/mol) showed comparatively higher potency against AD and PD associated targets than currently used standard drugs. Overall, the potential binding affinity from molecular docking, static thermodynamics feature from MD-simulation and other multiparametric drug-ability profiles suggest that karanjin could be considered as a suitable therapeutic lead for AD and PD treatment. Furthermore, the present results were strongly correlated with the earlier study on karanjin in an Alzheimer’s animal model. However, necessary in vivo studies, clinical trials, bioavailability, permeability and safe dose administration, etc. must be required to use karanjin as a potential drug against AD and PD treatment, where the in silico results are more helpful to accelerate the drug development.

## 1. Introduction

Alzheimer’s disease (AD) is one of the most common neurodegenerative diseases and is the most popular cause of dementia in adults. AD is marked by behavioral changes, cognitive impairments and imperfection in conducting routine life tasks, overall creating a major socio-economic strain on the health care system [1,2]. It is reported that one new case of dementia is estimated to occur every three seconds in the world, where approximately 55 million individuals have dementia, affecting 60% in low- and middle-income countries [3]. In fact, there are about 10 million new cases reported every year, and the overall number of dementia patients is estimated to hit 82 million in 2030 and 152 million in 2050 [4]. In Malaysia, the Alzheimer’s Disease Foundation Malaysia [5] estimates that there are currently around 50,000 individuals affected by the disease, and, by 2030, the figure is expected to be 100,000 and will continue to grow to 250,000 in 2050 [5]. Pathophysiologic changes of the disease include deficiency in the essential neurotransmitter acetylcholine (ACh), accumulation of amyloid plaques (Aβ), heavily phosphorylated tau proteins and imbalances in the gluatamatergic system [6,7,8]. To date, only five drugs are clinically approved, including the cholinesterase inhibitors tacrine, galantamine, donepezil, rivastigmine and the glutamatergic system modulator memantine. Nevertheless, these medications have limited effectiveness with many associated side effects [9]. The availability of pre-clinical and clinical trials on mild to moderate AD dementia is timely for the development of more effective and safe natural alternatives [10].

**Figure 1:**
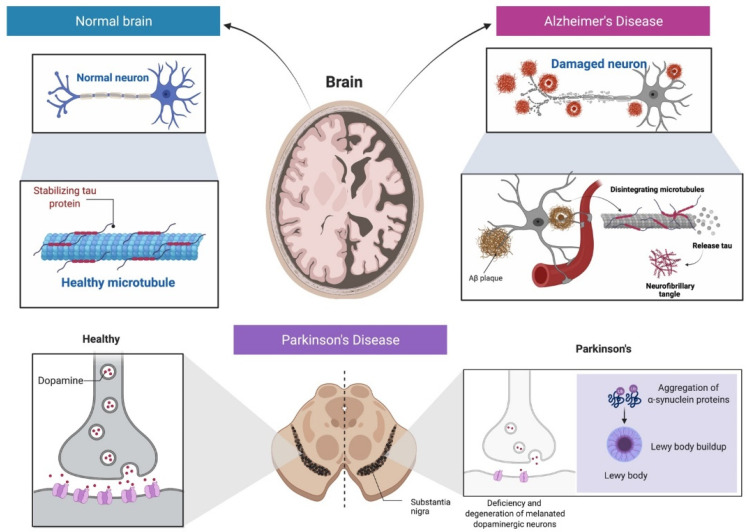
Pathological features of AD and PD (involving tau protein) to the healthy brain. PD is characterized by the accumulation of Lewy bodies (clusters of alpha synuclein protein coupled with ubiquitin) and the degeneration of dopaminergic neurons.

Parkinson’s disease (PD) is another common type of neurodegenerative disease affecting the nigrostriatal pathway in the brain as a result of by-product formation (Lewy bodies) and the lack of a dopamine neurotransmitter (Figure 1) [11]. In fact, the loss of dopaminergic neurons in the substantia nigra (SN) is a hallmark of PD, which affects 1–2% individuals above 60 years old [12]. According to projections, the disease causes 5–35 new cases per 100,000 individuals [13], with increasing frequency as age progresses [14]. The proportion of individuals affected by PD is rising rapidly, with predictions of a doubled number by 2030 [15]. Variations in the motor response oscillations generated concurrently with drug-induced dyskinesia commonly seen in about one-third of PD patients following three to six years of drug use is a profound drawback in current treatments of PD [16]. To date, carbidopa, levodopa, anticholinergics, dopamine agonists, monoamine oxidase B inhibitors, catechol-o-methyltransferase inhibitors and amantadine are all used in the treatment of PD, with the most common being levodopa [17,18]. These medications have numerous side effects and frequently result in further complications [19]. Therefore, there is an urgent need to search for novel medicinal agents with minimal adverse effects, where identification of such active phytochemical(s) may be considered as an ideal approach to counter both AD and PD, significantly.

**Figure 2:**
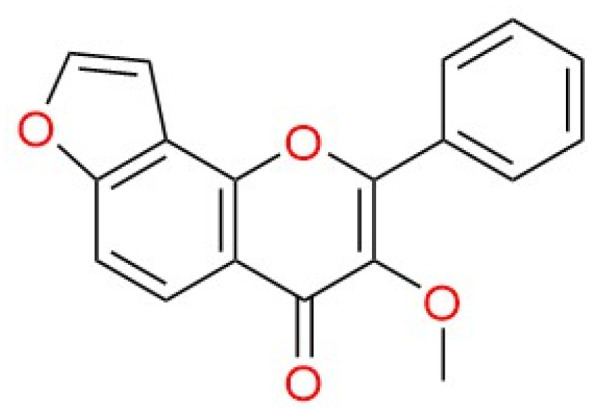
Chemical structure of karanjin (3-methoxy-2-phenylfuro[2,3-h]chromen-4-one).

Despite the limited success of synthetic agents as potential multifunctional drugs against AD and PD, the main limiting factors such as pharmacokinetics and safety issues remain challenging [9]. Unfortunately, currently available medications provide only symptomatic relief and do not stop neurodegeneration, making novel drug discoveries important. Natural products can potentially offer effective and safe pharmacodynamic characteristics in challenging neurodegenerative diseases. Nevertheless, the number of biological pathways and proteins implicated in the diseases’ pathogenesis, the complexity of the affected organs (especially the brain) and their aggressiveness remain the biggest obstacles toward drug development for AD/PD [20,21]. Plant-derived natural products, as well as their bioactive molecules, have been widely researched in recent years for their therapeutic potential in a range of neurodegenerative diseases including AD and PD [22,23]. Among numerous molecules, flavonoids are slated to have an excellent neuroprotective profile based on the findings of several works of epidemiological research [24]. Karanjin (3-methoxy-2-phenylfuro[2,3-h]chromen-4-one) is a furanoflavonoid (Figure 2), obtained mainly from *Pongamia pinnata* (L.) Pierre (family: Fabaceae). It is well known for its wide range of biological activities including antioxidant, anticancer, antidiabetic, anti-inflammatory and anti-ulcer [25]. In addition, karanjin has also been investigated in a behavioral study against Alzheimer’s, where the experimental animals demonstrated a progression of improved memory in AD-induced animals [26]. Hence, karanjin is a promising agent in the management of neurodegenerative diseases including its prevention and treatment, contributed by its antioxidant effect [27,28]. Apart from that, no studies have reported on the use of karanjin against neurodegenerative disorders, especially against AD and PD.

The molecular docking study is a computational-based study used to investigate the potency of any derived candidate at a primary stage, targeting any disease-associated target. Currently, most researchers use advanced computational tools during ‘hit’ or ‘lead’ candidate selection [29,30,31]. Indeed, natural products or phytochemicals tend to contain multi-potent biological activities. Therefore, evaluating individual potencies in a random experimental study is a complex and time-consuming procedure. In this situation, molecular docking is a more suitable approach to assess the strength of any desired natural products before conducting a randomized experimental study. In fact, to date, molecular docking is considered as an advanced and cost-effective technique to avoid the random practical or ‘hit-and-trial’ method of drug screening [29,31,32]. However, molecular docking is an early guidance tool in contemporary drug discovery to minimize the time-resource and due to the fact that drug candidates for human use cannot be recommended in the absence of extensive experimental and pharmacological studies. Overall, molecular docking is user-friendly and is a good potential tool in drug development. Scientific evidence shows that the prediction results based on in silico studies are comparable with in vitro and in vivo results [33]. In this study, a detailed in silico molecular docking investigation was conducted on karanjin with several protein targets in relation to AD and PD for a new drug design and development. To clarify information on their thermodynamic and dynamic properties, as well as to confirm the docking results, molecular dynamics simulations were performed on karanjin, followed by the calculation of the binding free energy. Furthermore, to ensure karanjin’s safety and efficacy in the treatment of AD and PD, its physicochemical, drug-likeness and ADMET profiles were also studied.

## 2. Results and Discussion

### 2.1. Physicochemical, Drug-Likeness and ADMET Properties of Karanjin

Karanjin appears to follow all five of Lipinski’s drug-likeness criteria (Table 1). According to the data acquired from DruLiTo software, karanjin also passed Veber’s rule, the blood-brain barrier (BBB) likeness rule, unweighted quantitative estimate of drug-likeness (QED) and weighted QED, but it failed the Ghose filter, CMC-50 like rule and molecular detection of rug resistance (MDDR) like rule. All of the above findings indicate that it is a good potential drug-like molecule and a useful therapeutic agent against a variety of disorders including neurodegenerative disorders.

The ADMET properties of karanjin were evaluated using the online software vNN-ADMET webserver. The results were presented as both restricted and unrestricted prediction models. Karanjin did not exhibit drug-induced liver injury and cytotoxicity according to the unrestricted prediction model of the software, whereas there was no high confidence prediction available with the restricted model. Karanjin may produce positive results for human liver microsomal stability assay, and it may be rapidly metabolized according to the unrestricted applicability domain. Additionally, karanjin may produce the inhibition of CYP 1A2, 3A4, 2D6, 2C9 and 2C19, as predicted by both models. Karanjin can cross the BBB and may also cross the membrane transporter. Karanjin may inhibit p-glycoprotein. Subsequently, the half-lives of substrates increase due to reduced billiary excretion and clearance of the substrates in the kidney proximal tubule, thereby enhancing renal reuptake [34]. Based on the Ames Mutagenicity assay, karanjin does not cause mutation. According to the mitochondrial toxicity assay, it also does not produce mitochondrial dysfunction. The MRTD for karanjin was established to be 157 mg/day. All of the ADMET results and drug-likeness attributes were consistent with those yielded from other tools, such as SwissADME and admetSAR. Overall, the ADMET characteristics indicate that karanjin is safe for therapeutic use.

### 2.2. In Silico Results of Karanjin against AD and PD

**Figure 3:**
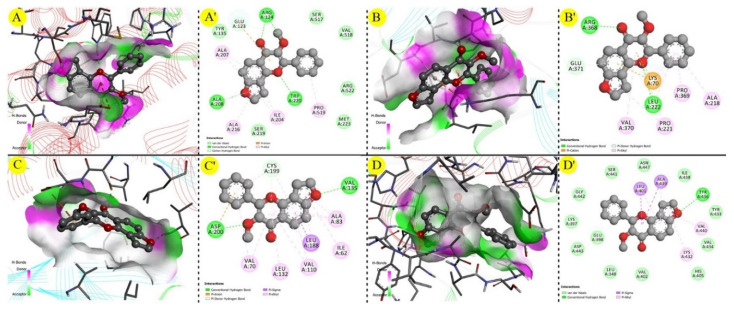
Molecular interactions of the natural karanjin against four putative drug targets of PD during study docking in 3D and 2D views (AutoDock); (**A**,**A′**) interaction against ACE (PBD ID: 1O86); (**B**,**B′**) interaction against BACE1 (PBD ID: 4DJU); (**C**,**C′**) interaction against GSK-3 (PDB ID: 1Q5K); and (**D**,**D′**) interaction against TACE (PDB ID: 2OI0), respectively. The 3D and 2D images were generated using the software Discovery Studio Visualizer. The grey spheres represent the ligand (karanjin), and arrows indicate the interaction of the karanjin with the amino residues present in the protein. The green colored line indicates the hydrogen bond interaction with the amino acid residues.

The individual ligands docking score against individual targets were recorded in Table 2. As per the AutoDock software, the docking score is always expressed in a negative value, where a higher negative value indicates a better potency. Karanjin exhibited a docking score within −7 to −10 kcal/mol against the selected four AD-associated targets. The highest potency was seen against TACE (PDB ID:2OI0), with a docking score of −9.16 kcal/mol, while the lowest was against ACE (PDB ID: 1O86), with a docking score of −7.54 kcal/mol (Table 2 and Figure 3). Similarly, standard drugs exhibited docking scores within −5 to −11 kcal/mol against five AD-associated targets. In contrast, donepezil exhibited a higher docking score (−11.0 kcal/mol) against TACE, while rivastigmine showed a lower docking score (−5.31 kcal/mol) against GSK-3. The progression of AD involves the destruction of the cholinergic neurons in the brain, since most of the palliative treatments for AD involve the use of cholinesterase inhibitors (ChEIs) including donepezil, rivastigmine and galantamine that impede the action of acetylcholinesterase (AChE), which hydrolyzes acetylcholine (ACh). The main therapeutic approach in dealing with AD is via the enhancement of cholinergic neurotransmission by preventing one of the major neurotransmitters, ACh from being broken down by AChE, which in turn maintains the brain’s ACh level to compensate for the loss of functioning brain cells. In AChE (PDB ID: 6ZWE), the investigated standard drugs exhibited docking scores between −7.3 to −11.0 kcal/mol, while karanjin showed a better docking score of −9.4 as compared with rivastigmine and galantamine. Overall, the results indicated that karanjin exhibited a comparatively similar potency to the standard drugs. These findings are well associated with those yielded from the Molegro Virtual Docker program as well.

Neuro-inflammation is linked to several neurodegenerative disorders, including AD. Normally, the levels of tumor necrosis factor-α (TNF-α) are maintained at relatively low levels, but, as AD progresses, the levels rise [35,36] The interactions of karanjin with TACE were similar to those described in previous works of research with docking results of other molecules [37]. ACE inhibition is a prospective treatment target for AD because angiotensin II can impair memory consolidation in several investigations [38,39]. The key interactions of karanjin with ACE were discovered and were closely associated with those reported in previous investigations [40]. The inhibition of BACE-1 is becoming more widely recognized as a possible therapeutic method for the drug development of AD.

BACE-1 has a broad substrate-binding domain with an affinity for a variety of substrates, making the discovery of small molecule inhibitors that can occupy such a large size more challenging [41]. GSK3 is over-expressed in the brains of AD patients, thus contributing to tau protein hyperphosphorylation and AD development [42]. Thus, inhibition is becoming a very promising therapeutic technique in the treatment of AD [42]. Currently, only three AChE inhibitors (donepezil, rivastigmine and galantamine) are utilized in AD therapy. However, these drugs only provide symptomatic relief and are generally used to treat mild to moderate dementia [20]. The molecular interactions of karanjin were closely linked with the docking data for other natural/synthetic compounds with BACE-1 [43,44,45,46,47,48] and AChE [49,50,51], as previously reported.

**Figure 4:**
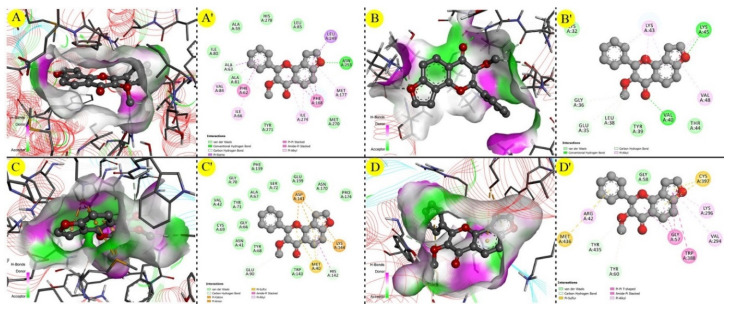
Molecular interactions of the natural karanjin against four putative drug targets of AD during study docking in 3D and 2D views (AutoDock); (**A**,**A′**) interaction against A_2A_AR (PDB ID: 3EML); (**B**,**B′**) interaction against ASN (PDB ID: 1XQ8); (**C**,**C′**) interaction against COMT (PDB ID: 1H1D); and (**D**,**D′**) interaction against MAO_B (PDB ID:2C65), respectively. The 3D and 2D images were generated using the software Discovery Studio Visualizer. The grey spheres represent the ligand (karanjin), and the arrows indicate the interaction of the karanjin with the amino residues present in the protein. The green colored line indicates the hydrogen bond interaction with the amino acid residues.

**Figure 5:**
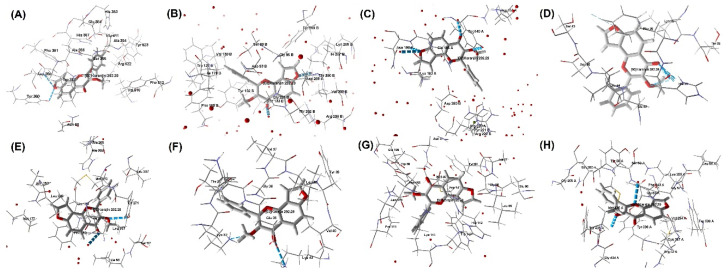
Molecular interactions of the natural karanjin against four putative drug targets for each AD and PD (Molegro Virtual Docker); (**A**) interaction against ACE (PBD ID: 1O86); (**B**) interaction against BACE1 (PBD ID: 4DJU); (**C**) interaction against GSK-3 (PDB ID: 1Q5K); (**D**) interaction against TACE (PDB ID: 2OI0); (**E**) interaction against A_2A_AR (PDB ID: 3EML); (**F**) interaction against ASN (PDB ID: 1XQ8); (**G**) interaction against COMT (PDB ID: 1H1D); and (**H**) interaction against MAO_B (PDB ID:2C65), respectively. The Blue colored lines in the figure indicate hydrogen bond interaction.

On the other hand, karanjin displayed a docking score between −4 and −10 when compared against the four selected PD-associated targets (Table 2), where it was found to be higher against MAO_B (PBD ID: 2C65) (docking score: −9.22 kcal/mol) and lower against ASN (PDB ID: 1XQ8) (Table 2 and Figure 4). Similarly, all three standard drugs exhibited docking score values of between −4 and −10 kcal/mol, where rasagiline showed a higher docking score (−8.44 kcal/mol) against COMT (PDB ID: 1H1D) and a lower docking score (−4.23 kcal/mol) against ASN (PDB ID: 1XQ8). Thus, overall, karanjin has a higher potency against PD-associated targets as compared to standard drugs. These observations were confirmed with the findings from the Molegro Virtual Docker program (Table 2 and Figure 5).

The activity of non-dopaminergic A2A receptor antagonists has been reported in several previous investigations, making them a promising target for the development of anti-Parkinson drugs [52,53], where a doubling or tripling of the α-synuclein gene has been linked to a similar form of PD [54,55]. In humans, MAO can be expressed in two different isoforms: MAO-A and MAO-B, which have different substrate affinities and tissue distributions. However, owing to its role in dopamine deamination in the brain, MAO-B is the principal pharmacological target in PD [56]. Additionally, as age progresses, the brain tends to contain higher MAO-B expression levels, which leads to increased dopamine metabolization and hydrogen peroxide formation, which accelerates dopaminergic neuronal cell death [56]. Since MAO-B is involved in dopamine metabolism, selective inhibitors of this enzyme may be useful in the treatment of PD.

In humans, COMT is expressed in two molecular isoforms: (1) soluble form (SCOMT) and (2) membrane-bound (MBCOMT), which is the main isoform in the brain [57]. COMT is a ubiquitous enzyme responsible for the O-methylation of catechol substrates such as dopamine. Owing to its role in dopamine and L-DOPA metabolisms, COMT is becoming increasingly associated with PD pathophysiology [57]. In fact, all of the target proteins had key interactions with karanjin. The interactions are similar to previous works of research with docking results of other bioactive substances with A2A [58,59], ASN [60,61], MAO-B [62,63,64] and COMT [65,66,67].

### 2.3. Molecular Dynamics Simulation Study

**Figure 6:**
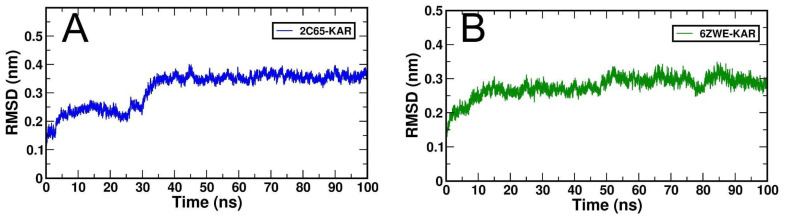
Generated RMSD plots for both docking complexes from MD simulation at 100 ns. (**A**), RMSD plot of 2C65-KAR and (**B**), RMSD plot of 6ZWE-KAR.

**Figure 7:**
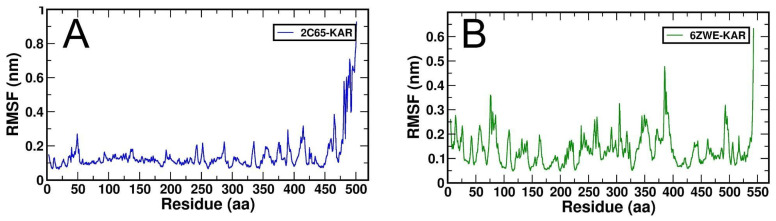
Generated RMSF plots for both docking complexes from MD simulation at 100 ns. (**A**), RMSF plot of 2C65-KAR and (**B**), RMSF plot of 6ZWE-KAR.

**Figure 8:**
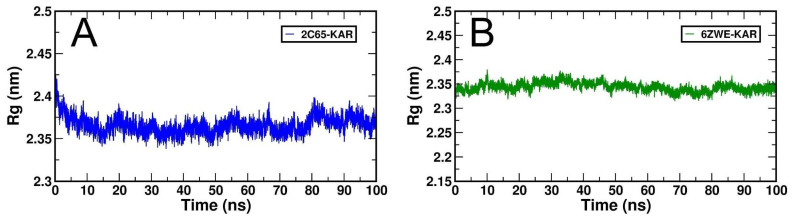
Generated Rg plots for both docking complexes from MD simulation at 100 ns. (**A**), Rg plot of 2C65-KAR and (**B**), Rg plot of 6ZWE-KAR.

**Figure 9:**
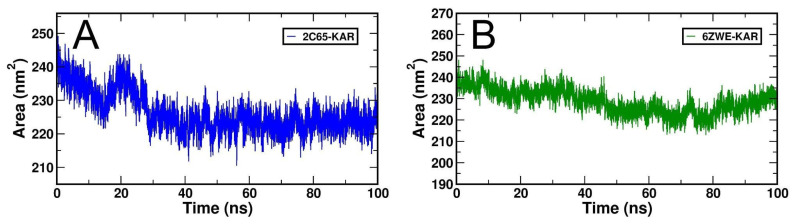
Generated SASA plots for both docking complexes from MD simulation at 100 ns. (**A**), SASA plot of 2C65-KAR and (**B**), SASA plot of 6ZWE-KAR.

**Figure 10:**
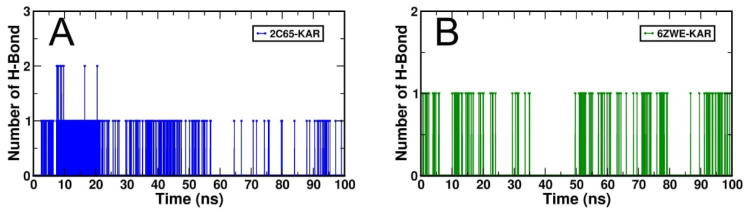
Generated H-bond interaction result for both docking complexes from MD simulation at 100 ns. (**A**), H-bond interaction plot of 2C65-KAR and (**B**), H-bond interaction plot of 6ZWE-KAR.

The dynamic behaviors or molecular stability of two selective docking complexes, 2C65-KAR and 6ZWE-KAR was observed through generated root-mean-square deviation (RMSD), Root mean square fluctuation (RMRF), radius of gyration (Rg), Solvent accessible surface area (SASA) and number of H-bond interactions with binding energy, individually by MD stimulation at 100 ns (Figure 6, Figure 7, Figure 8, Figure 9 and Figure 10; Table 3). From RMSD plots, the 2C65-KAR complex initially deviated up to 35 ns within the range of 0.1 to 0.25 nm and further gained stability till 100 ns (Figure 6A). Similarly, in the 6ZWE-KAR complex, a higher fluctuation was found with 75–85 ns at a range of 0.2 to 0.3 nm (Figure 6B). Overall, karajin maintained its interaction stability with both targets, but a little bit of deviation was observed throughout 100 ns.

RMSF plots of both docking complexes were generated, where 2C65-KAR complexes showed a static feature (Figure 7A) and 6ZWE-KAR complex is a destabilized form (Figure 7B). The RMSF plot expresses the amino acids residual fluctuation of a protein during interaction with a ligand at a particular time scale or ns. Overall, both RMSF plots indicated that karanjin interacted in a stabilized form with 2C65 target throughout 100 ns.

The compactness of the native protein can be determined by the generated Rg-plots. Folding and unfolding of the protein was analyzed by the RG-values at 100 ns time scale for 2C65-KAR and 6ZWE-KAR docking complexes (Figure 8A,B). However, the 2C65-KAR docking complex Rg-plot is comparatively less stable than 6ZWE-KAR.

Similarly, the SASA plots indicated the compactness of in form of native constant of the target protein after interaction with an inhibitor mainly hydrophobic interaction. Therefore, the SASA plots were calculated for both docking complexes of karanjin against, 2C65 AND 6ZWE, individually. However, both complexes are deviated throughout 100 ns (Figure 9). From a minute observation, 2C65-KAR gained stability after 25 ns (Figure 9A), but 6ZWE-KAR continuously deviated in SASA values (Figure 9B).

Overall, each docking complex was stabilized through strong H-bond interactions, with 2C65-KAR (Figure 10A) and 6ZWE-KAR (Figure 10B) also stabilized through H-bonds interactions with both targets.

Similarly, distinguished dynamic behaviors of both targets were observed through the widely accepted molecular mechanics Poisson-Boltzmann (MMPBSA) methods, and the results of binding energy of 2C65-KAR and 6ZWE-KAR were −161.262 kJ/mol and −168.652 kJ/mol, respectively (Table 3). Thus, the compactness and stability of karinjin with both targets are comparatively similar at 100 ns time scale.

### 2.4. Frontier Molecular Orbitals (FMOs) and Density Functional Theory (DFT) Analyses

**Figure 11:**
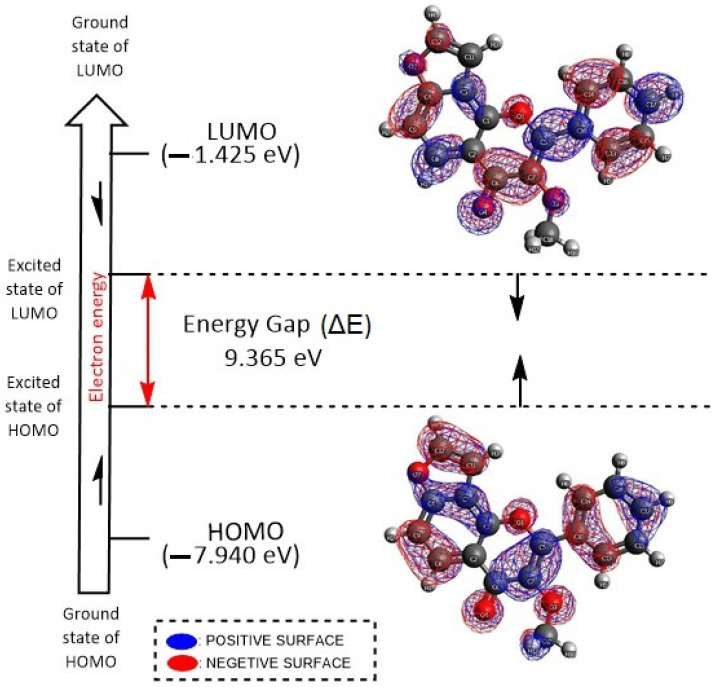
Frontier molecular orbitals (FMOs) analysis of karanjin in the form of LUMO, HOMO and their energy gap (ΔE eV) using the software Avogadro.

Based on the generated highest occupied molecular orbital (HOMO) (−7.940 eV), the lowest unoccupied molecular orbital (LUMO) (−1.425 eV) and the higher energy gap (ΔE = 9.365 eV) indicated that a higher charge transmission occurs within karanjin and forms a stable interaction with the target protein towards enhancement of the bioactivity (Figure 11). The ΔE value indicates that the molecule has strong inhibition efficiency because the energy required to remove an electron from the last occupied orbital is minimized. Excellent molecule inhibitors accept free electrons as well as donate electrons to a vacant orbital, making them more electron-rich and thus offering superior inhibition efficiency. Overall, the present results demonstrated that karanjin is rich in electrons, since the LUMO value indicates a molecular species’ potential to receive free electrons and provide good inhibition performance.

Overall, the current in silico findings are strongly associated with karanjin’s anti-AD’s activity as measured by the in vivo diazepam-induced amnesia in mice elevated by plus maze and Morris water maze models [26]. In mice, oral administration of karanjin (50 mg/kg, p.o.) significantly reversed diazepam-induced amnesia, indicating improved learning and memory, and exhibited anti-AD activity similar to that reported for the standard drug piracetam (200 mg/kg). Apart from the aforementioned study, no other in vitro or in vivo investigations on karanjin’s anti-AD and anti-PD or even anti-Huntington’s disease effects have been reported. Nevertheless, the docking score and molecular interactions supported the fact that karanjin is a potential neuroprotective candidate.

## 3. Materials and Methods

### 3.1. Physicochemical and Drug-Likeness Properties of Karanjin

The physicochemical properties of karanjin were mainly obtained from PubChem [68], since understanding the molecule’s physicochemical properties is the first step to allow it to be transformed into a drug-like molecule. The drug-likeness properties (molecular weight, H-bond donors, H-bond acceptors, log P value and rotatable bonds) as described in Lipinski’s rule of five were calculated using Biovia Discovery Studio 19.0 (http://www.niper.gov.in/pi_dev_tools/DruLiToWeb/DruLiTo_index.html (accessed on 27 December 2021), offline open-source software) [69]. Overall, compounds that do not breach Lipinski’s rule of five may have better folding, polarity and molecular size and are expected to have a more promising therapeutic effects [70].

### 3.2. ADMET Properties of Karanjin

The vNN-ADMET webserver was used to predict the ADMET properties (http://www.swissadme.ch/, accessed on 18 December 2021) [28]. Along with that, other parameters include liver toxicity, metabolism (cytochrome P450 or CYPs 1A2, 3A4, 2D6, 2C9 and 2C19 to develop CYP inhibition), membrane transporters, human ether-a-go-go-related gene (hERG) for the evaluation of cardiotoxicity, mitochondrial toxicity (MMP), mutagenicity (AMES test) and the maximum recommended therapeutic dose (MRTD).

### 3.3. In Silico Study of Karanjin against AD and PD

The docking approach involved predicting the conformation and orientation of ligands within a targeted binding site. In general, docking investigations have two objectives: (1) accurate structural modelling and (2) correct activity prediction. Docking is usually thought of as a multi-stage process, with each step adding one or more levels of complexity. The procedure starts with the use of docking algorithms to position small molecules in the active site. These algorithms are supplemented with scoring functions that evaluate interactions between molecules and potential targets in order to predict biological activity.

Five human protein targets associated with AD and four human protein targets associated with PD were chosen to investigate karanjin’s neuroprotective effects based on an in silico molecular docking approach. Table 4 summarizes the protein targets and the criteria for selection used in the present investigation. As per the requirements, the retrieved three-dimensional (3D) crystal structure of selected targets was from the protein data bank (PDB) [71] with individual PDB IDs.

AD-associated targets such as angiotensin converting enzyme (ACE) with PBD ID: 1O86_A), β-site APP cleaving enzyme 1 (BACE1) with PDB ID: 4DJU_A, glycogen synthase kinase-3 (GSK-3) with PDB ID: 1Q5K_A, TNF-α converting enzyme (TACE) with PDB ID: 2OI0_A and acetylcholinesterase (AChE) with PDB ID: 6ZWE were used. Similarly, PD-associated targets, A2A adenosine receptor (A_2A_AR) with PDB ID: 3EML_A, α-synuclein (ASN) with PDB ID: 1XQ8_A, catechol-O-methyltransferase (COMT) with PDB ID: 1H1D_A and monoamine oxidase B (MAO-B) with PDB ID: 2C65_A were incorporated. Furthermore, we have selected three standard drugs, each against AD (donepezil, galantamine and rivastigmine) and PD (dopamine, rasagiline and selegiline), as a ligand for the computational based investigation [33].

Then, the 3D-chemical structure of both karanjin and six standard drugs was retrieved from the PubChem database [68]. As per docking software, both target and ligand structures were saved in dot PDB (.pdb) file format for a docking study using the software AutoDock 4.1 (https://autodock.scripps.edu/ (accessed on 25 November 2021), offline open-source software) [72,73]. The Discovery Studio visualizer software was used for molecular interaction of generated protein–ligand complexes during the docking study [72,73]. The molecular docking investigation was conducted using Molegro Virtual Docker 6.0 in addition to AutoDock 4.1, and the findings were compared (http://molexus.io/molegro-virtual-docker/ (accessed on 21 November 2021), MVD 2013.6.0.1–2013-12-13 academic license).

### 3.4. Molecular Dynamics Simulation Study

Molecular dynamics simulations were performed on selected protein-ligand complexes such as 2C65-Karnajin (2C65-KAR) and 6ZWE-Karnajin (6ZWE-KAR) using Gromacs-2019.4 (http://www.gromacs.org/ accessed on 15 January 2022). The selected ligand topology was downloaded from the PRODRG server (PMID: 15272157) to obtain the force field coordinates. The steepest descent algorithm was used to prepare the system, and vacuum was minimized for 1500 steps. The complex structures in a cubic periodic box of 0.5 nm were solvated using a simple point charge (SPC) water model. Adding sufficient numbers of Na^+^ and Cl^−^ counter ions was then sufficient to maintain the complex system with a salt concentration of 0.15 M. Based on a literature review, the system preparation was discussed. A final simulation run of 100ns was conducted in the ensemble after the NPT equilibration phase (PMID: 31514687). GROMACS simulation package was used to analyze the Protein RMSD, RMSF, RG, SASA and H-Bond (PMID: 32567989). The Molecular Mechanics Poisson-Boltzmann Surface Area (MM-PBSA) method was used during calculation of binding free energy (ΔG binding) calculation of simulated docking using the GROMACS utility g_mmpbsa suit. Overall, the results were obtained by computing ΔG in the last 50 ns within 1000 frames (PMID: 24850022).

### 3.5. FMOs and DFT Analyses

We have employed DFT and FMOs analyses to explore chemical space distribution of karanjin structure in the form of HOMO, LUMO and their energy gap (ΔE), Mulliken population and electric charge distribution using the software Avogadro-ORCA 1.2.0 [92,93]. Primarily, the karajin structure was optimized/energy minimized using Universal Force Field (UFF) with the steepest descent algorithms; then, the single-point energy calculation with the restricted Hartree–Fock (RHF) principle in the Cartesian format was employed to compute the electron HOMO, LUMO and ΔE orbital energies [92,93]. Similarly, DFT analyses were carried out using Becke’s three parameters, Lee-Yang-Parr exchange correlation functional (B3LYP) and a balanced polarized triple-zeta basis set, def2-TZVP, with all default parameter settings in Avogadro-ORCA 1.2.0 [92].

## 4. Conclusions and Future Perspectives

The in silico findings on karanjin indicated that it has potential neuroprotective properties due to its ability to bind to specific protein targets for AD and PD. Karanjin exhibited comparatively higher than standard drugs used against both AD and PD. The information on thermodynamic and dynamic properties of karanjin was clarified by molecular dynamics simulations, which were connected with the docking findings. Our study can serve as a basis for the development of a novel drug from a natural product which is cost effective for the treatment of neurodegenerative diseases. Moreover, the mechanism(s) of action of karanjin in preventing AD and PD progression can yield new knowledge in drug development for neurodegenerative disorders.

**Figure 12:**
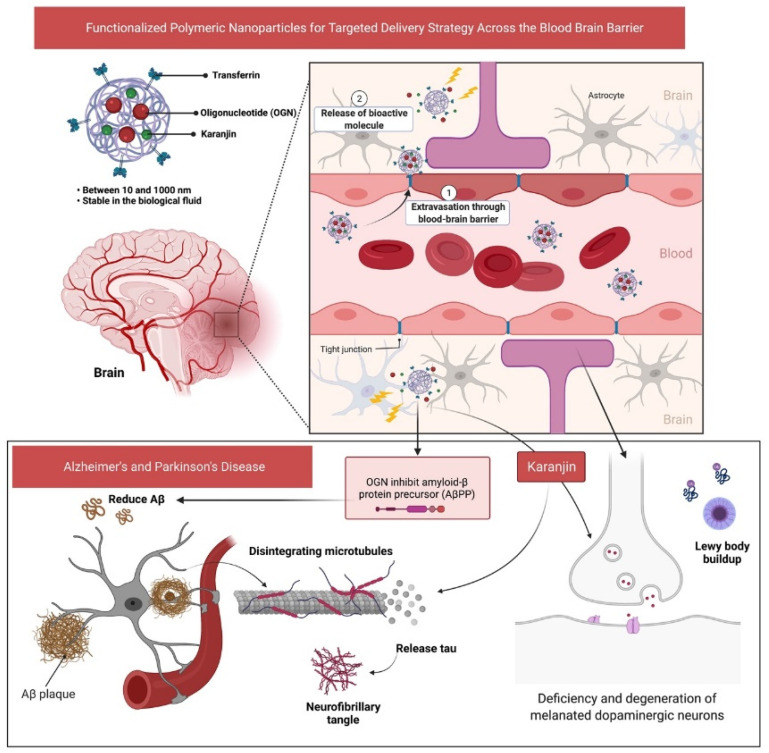
Schematic illustration of the proposed future perspective by employing surface modified polymeric nanoparticles loaded with oligonucleotides (OGN) and karanjin for enhanced delivery across the blood–brain barrier.

Polymeric nanoparticles present an appealing medium for therapeutic cargo delivery. Using standard and economically viable emulsion processes, polymers can be formed into nanoparticles with selected properties. Polymers that degrade and allow for ‘on-demand’ drug release by various internal physiological factors such as temperature and pH, as well as external stimuli including light and ultrasound, are more favorable for smart in situ targeting techniques. We believe that more progress may be made in future years toward developing techniques to halt the progression of neurodegenerative diseases. As for the future perspective, the use of a combination of oligonucleotides (OGN) and karanjin loaded in surface-modified polymeric nanoparticles can serve as enhanced delivery across the blood-brain barrier (Figure 12). The use of naturally occurring proteins, such as transferrin, as the targeting ligand is believed to bypass biological barriers such as the BBB, whereas OGN is said to reduce amyloid-β protein (Aβ) production. Furthermore, early prediction of the use of karanjin against AD and PD as shown in this study may help facilitate further development of the molecule for novel drug design and development. For building a “compound-protein/gene-disease” network and disclosing the regulating principles of small molecules in a high-throughput manner, network pharmacology is more successful. This method is very useful for analyzing medication combinations, particularly small compounds derived from natural materials. Such research will provide more evidence in the future to help with drug discovery and the development of Karanjin. Nevertheless, more in vitro and in vivo studies, as well as pharmacokinetics and bioavailability studies, structural modifications and structure activity relationships (SAR), are required. These steps can help confirm the prediction to enable the further development of karanjin as a drug molecule in the near future.

## Figures and Tables

**Table 1:** Physicochemical and drug-likeness properties of karanjin.

Property	Result (vNN-ADMET, swissADME and admetSAR Tools)
Molecular formula	C_18_H_12_O_4_
Molecular weight	292.30
Hydrogen bond donors	0
Hydrogen bond acceptors	4
Rotatable bonds	2
*Log P* (Partition coefficient, Predicted value)	2.54 or 3.43
Melting point	157–159 °C (in the crystallized form)
Molar refractivity	81.027 cm^3^ or 84.18 cm^3^
Molar volume	214.875 cm^3^
Topological polar surface area	48.7 Å^2^ or 52.58 Å^2^
Lipinski’s rule of five	Passed
Ghose filter	Passed
Veber’s rule	Passed
BBB likeness rule	Passed
Unweighted QED	Passed
Weighted QED	Passed
GI absorption	High
BBB Permeant	Yes
CYP1A2, CYP2C19, CYP2C9, CYP2D6 and CYP3A4 inhibitors	Yes
Bioavailability score	0.55

Abbreviations: BBB, Blood Brain Barrier; QED, Quantitative Estimate of Drug-likeness.

**Table 2:** Molecular docking score (kcal/mol) of karanjin with three standard drugs against four different human targets proteins associated with AD and PD.

**Karanjin/Standards**	**Selected Targets Associated with AD**									
	**ACE** **(PBD ID: 1O86)**		**BACE1** **(PBD ID: 4DJU)**		**GSK-3** **(PDB ID: 1Q5K)**		**TACE** **(PDB ID: 2OI0)**		**AChE** **(PDB ID: 6ZWE)**	
	AutoDock	Molegro Virtual Docker	AutoDock	Molegro Virtual Docker	AutoDock	Molegro Virtual Docker	AutoDock	Molegro Virtual Docker	AutoDock	Molegro Virtual Docker
Karanjin	−7.54	−85.48	−8.79	−77.11	−8.23	−69.63	−9.16	−1289.34	−9.40	−107.87
Donepezil *	−8.88	−120.35	−9.21	−68.34	−7.69	−106.51	−11.00	−1642.78	−11.00	−80.81
Galantamine *	−7.42	−100.24	−7.06	−96.06	−6.68	−79.57	−8.48	−1096.31	−8.20	−108.12
Rivastigmine *	−6.47	−84.66	−6.66	−86.59	−5.31	−65.71	−7.57	−1191.48	−7.30	−98.58
	**Selected Targets Associated with PD**									
	**A_2A_AR** **(PDB ID: 3EML)**		**ASN** **(PDB ID: 1XQ8)**		**COMT** **(PDB ID: 1H1D)**		**MAO_B** **(PDB ID: 2C65)**		**--**	
	AutoDock	Molegro Virtual Docker	AutoDock	Molegro Virtual Docker	AutoDock	Molegro Virtual Docker	AutoDock	Molegro Virtual Docker	--	--
Karanjin	−8.39	−88.37	−4.75	−83.35	−8.95	−90.88	−9.22	−145.14	--	--
Dopamine *	−5.69	−59.07	−5.16	−67.09	−7.36	−87.40	−6.59	−82.62	--	--
Rasagiline *	−6.89	−72.66	−5.51	−66.95	−8.44	−104.91	−7.57	−97.88	--	--
Selegiline *	−5.53	−62.34	−4.23	−70.15	−7.56	−106.52	−6.98	−95.33	--	--

Notes: * Standard drugs; Abbreviations: ACE, Angiotensin converting enzyme; BACE1, β-site APP cleaving enzyme 1; GSK-3, Glycogen synthase kinase-3; TACE, TNF-*α* converting enzyme; A_2A_AR, A2A adenosine receptor; ASN, *α*-synuclein; COMT, catechol-O-methyltransferase; and MAO_B, monoamine oxidase B.

**Table 3:** MMPBSA results of Binding energy of 2C65 and 6ZWE.

Protein Code	Van der Waal Energy kJ/mol	Electrostatic Energy kJ/mol	Polar Solvation Energy kJ/mol	Binding Energy kJ/mol
2C65	−205.968	−4.908	66.203	−161.262
6ZWE	−197.955	−2.742	49.001	−168.652

**Table 4:** Targets in AD and PD.

Disease	Targets	Reason for Selected Targets	References
AD	ACE	It has been shown to block memory consolidation in some investigations.	Li and Buxbaum [74] Kölsch et al. [38] Monastero et al. [39] Fridman et al. [75]
	BACE1	BACE1, a *β*-secretase involved in the formation of *β*-amyloid peptide, which is a dominant component in AD.	Vassar [76] Koelsch [77] Ridler [78] Bao et al. [79]
	GSK3	GSK3 phosphorylates the Tau protein, whose expression is associated to AD.	Eldar-Finkelman and Martinez [80] Bhat et al. [81] Wang et al. [82] Kremer et al. [83]
	TACE	TNF-*α* is normally kept at relatively low levels, but, as AD progresses, the levels rise.	Chang et al. [35] Dickson [84] Cheng et al. [36] Zhou and Bickler [85]
	AChE	AChE inhibition may affect amyloid precursor protein processing and protect neurons against a variety of insults.	Rees and Brimijoin [86]
PD	A_2A_AR	The basal ganglia have a more selective and extensive distribution of A_2A_. This selective receptor distribution may help to ensure fewer side effects, making nondopaminergic antagonists against PD.	Wilson and Mustafa [87]
	ASN	PD is caused by a doubling or tripling of the α-synuclein.	Olanow and Brundin [88] Chartier-Harlin et al. [54] Ibanez et al. [55]
	COMT	The COMT gene codes for an enzyme which degrades catecholamines, and this process is slowed in people with PD.	Martínez-Jauand et al. [89]
	MAO-B	MAO-B expression has been found in human brains, specifically in the substantia nigra of patients with PD.	Teo and Ho [90] Choi et al. [91]

## Data Availability

The data presented in this study are available on request from the corresponding author.
